
JOURNAL INFORMATION
==============================
NLM Title Abbreviation: ScientificWorldJournal
Journal ID: ScientificWorldJournal
NLM Journal ID: 101131163
Journal ID: TSWJ

The Scientific World Journal

ISSN: 2356-6140
EISSN: 1537-744X
Publisher: Wiley

ARTICLE INFORMATION
==============================
PMCID: PMC6009287
PMID: 29973801
DOI: 10.1100/tsw.2002.27
Article ID: 439782
Article version: 1
Subjects: Short Report

Crystallographic Studies of Bacterial Exoribonucleases

Fiedler Tristan J.
Malhotra Arun
Department of Biochemistry & Molecular Biology, University of Miami School of Medicine, P.O. Box 016129, Miami, FL 33101-6129, USA
Publication date: 2002
Electronic publication date: 2002 Mar 16
Volume: 2
First page: 53
Last page: 54
Copyright: Copyright © 2002 Tristan J. Fiedler and Arun Malhotra.
Copyright year: 2002
License: This is an open access article distributed under the Creative Commons Attribution License, which permits unrestricted use, distribution, and reproduction in any medium, provided the original work is properly cited.
License URL: https://creativecommons.org/licenses/by/3.0/

Funding: Florida Biomedical Research Foundation BM030

==============================
Advances in Gene Technology: The Genome and Beyond –
Structural Biology for Medicine (Proceedings of the 2002 Miami
*Nature Biotechnology* Winter Symposium)
*TheScientificWorld* 2002, 2(S2), 53–54
ISSN 1532-2246; DOI 10.1100/tsw.2002.27

### CRYSTALLOGRAPHIC STUDIES OF BACTERIAL EXORIBONUCLEASES

Tristan J. Fiedler and Arun Malhotra
Department of Biochemistry & Molecular Biology, University of Miami School of Medicine, PO Box 016129, Miami, FL 33101-6129

**INTRODUCTION.** Ribonucleases (RNases) play a central role in all cellular RNA processes. These processes include mRNA degradation, and maturation and turnover of stable RNAs, which are vital for the proper functioning of all cells. *E. coli* has served as a model system for understanding the role of ribonucleases in RNA metabolism, and eight distinct exoribonucleases have been identified in this bacterium. Of these, three (RNase T, RNase D, and oligoribonuclease) are members of a larger exonuclease superfamily (named the DEDD exonuclease family, after the four invariant acidic residues in these proteins) that includes the proof-reading domains of DNA polymerases[1].

While these three proteins share similar sequence motifs, they are functionally quite different. RNase T is involved in end-turnover of tRNAs and the maturation of tRNAs, 23S, and 5S RNAs. Interestingly, RNase T has been shown recently to also be a potent Dnase[2], and may play a role in end-trimming reactions during DNA recombination and repair[3]. RNase D is also involved in the maturation of tRNAs and small RNAs, but mainly as a backup enzyme. RNase D functions as a monomer, unlike RNase T and oligoribonuclease that exist as dimers. Oligoribonuclease catalyzes the degradation of very short RNAs (4 residues or shorter). It is the only exoribonuclease essential for cell viability, and its absence leads to accumulation of small oligoribonucleotides derived from mRNAs[4].

We have initiated crystallographic studies of this family of bacterial exoribonucleases, in collaboration with the laboratory of Dr. Murray Deutscher at the University of Miami, to structurally characterize these proteins and understand their mechanisms of exoribonuclease activity at an atomic resolution. The long term goals of this research are to understand the structures and mechanisms of action of all the exoribonucleases in a single organism (*E. coli*); these studies will complement a parallel study to completely determine and characterize the physiological role of all the exoribonucleases in *E. coli*, now underway in the Deutscher laboratory.

**METHODS**. RNase T and oligoribonuclease were overexpressed and purified from *E. coli* using standard column chromatographic techniques. RNase T was crystallized using ammonium sulfate as a precipitant. Oligoribonuclease was crystallized using sodium citrate as a precipitant, using the hanging drop methodology. Selenomethionine labeled oligoribonuclease was also purified, and crystallized for MAD phasing. These crystals are in the orthorhombic spacegroup (P212121; a = 70.4, b = 72.9, c = 147.8 Å).

Diffraction data on oligoribonuclease were collected to 2.0 Å resolution on the X8c and X12b beamlines at the National Synchrotron Light Source, Brookhaven National Laboratory. For the selenomethionine labeled oligoribonuclease, we collected diffraction data at three wavelengths for multiple anomalous diffraction (MAD) phasing. Twenty SeMet sites (five in each of the four oligoribonuclease molecules) were located using *SOLVE*[5], and used to obtain an experimental map extending to about 2.2 Å. Model building is currently in progress.

**RESULTS.** We are currently building an atomic model for oligoribonuclease using experimental phases extending to 2.2 Å. Two dimers of oligoribonuclease can be seen in the asymmetric unit. The atomic structure and detailed structural analysis for this protein will be presented.

**ACKNOWLEDGEMENT.** This work was supported by a Florida Biomedical Research Foundation Award #BM030 (to A.M.).

**REFERENCES**

1. Zuo, Y. and Deutscher, M.P. (2001) Nucl. Acids Res. 29, 1–10.
2. Zuo, Y. and Deutscher, M.P. (1999) Nucl. Acids Res. 27, 4077–4082.
3. Ghosh, S. and Deutscher, M.P. (1999) Proc. Natl. Acad. Sci. U. S. A. 96, 4372–4377.
4. Viswanathan, M., Lanjuin, A., and Lovett, S.T. (1999) Genetics 151, 929–934.
5. Terwilliger, T. and Berendzen, J. (1999) Acta Crystallogr. D55, 849–861.
